# Better strategies for containing COVID-19 pandemic: a study of 25 countries via a vSIADR model

**DOI:** 10.1098/rspa.2020.0440

**Published:** 2021-04

**Authors:** Han Yan, Yuru Zhu, Jia Gu, Yaxuan Huang, Haoxuan Sun, Xinyu Zhang, Yuqing Wang, Yumou Qiu, Song Xi Chen

**Affiliations:** ^1^ Guanghua School of Management, Peking University, Beijing, People’s Republic of China; ^2^ Center for Statistical Science, Peking University, Beijing, People’s Republic of China; ^3^ Yuanpei College, Peking University, Beijing, People’s Republic of China; ^4^ Center for Data Science, Peking University, Beijing, People’s Republic of China; ^5^ School of Mathematical Science, Peking University, Beijing, People’s Republic of China; ^6^ Department of Statistics, Iowa State University, Ames, IA, USA

**Keywords:** epidemic differential equations, policy effects, reproduction number, scenario analysis, varying coefficient model

## Abstract

We study epidemiological characteristics of 25 early COVID-19 outbreak countries, which emphasizes on the reproduction of infection and effects of government control measures. The study is based on a vSIADR model which allows asymptomatic and pre-diagnosis infections to reflect COVID-19 clinical realities, and a linear mixed-effect model to analyse the association between each country’s control measures and the effective reproduction number *R*_*t*_. It finds significant effects of higher stringency measures in lowering the reproduction, and a significant shortening effect on the time to the epidemic turning point by applying stronger early counter measures. Epidemic projections under scenarios of the counter measures (China and Korea, the USA and the UK) show substantial reduction in the epidemic size and death by taking earlier and forceful actions. The governments’ response before and after the start of the second wave epidemics were alarmingly weak, which made the average duration of the second wave more than doubled that of the first wave. We identify countries which urgently need to restore to at least the maximum stringency measures implemented so far in the pandemic in order to avoid even higher infection size and death.

## 1. Introduction

The Corona Virus Disease 2019 (COVID-19) caused a pandemic with more than 71 million infections and more than 1 million deaths worldwide [1] on 31 December 2020. By the end of April 2020, three months after Wuhan locked-down, there were more than 25 countries which had endured at least four weeks of community infections with good amount of epidemic data accrued [2–4]. Since then, 24 (14) of the 25 countries had experienced the second (third) wave of infections. Given the unprecedented global health crisis, there is an urgent need to estimate the infection rates of these countries, to learn from their epidemic paths, and to evaluate the effectiveness of their COVID-19 counter measures. Such an analysis would provide insights for choosing the necessary level of containment measures to counter the 2020–2021 winter pandemics of COVID-19. The effects of early control measures taken in China have been extensively studied, for example, [5–7] on public health interventions and control strategies on Wuhan’s outbreaks, [8,9] on both the domestic and international implications of the Wuhan travel ban; [10] on the transmissibility and severity in mainland Chinese locations outside Hubei.

Our evaluation is based on an extended SIR model [11] with time-varying coefficients [12]. Different from the classical SIR model, there are two pathways from the Infection compartment in the proposed model: the Asymptomatic pathway and the pre-symptomatic Infection pathway leading to the Diagnosed compartment, and hence is called the vSIADR model. The model permits infections in both pathways of disease progression. The pre-symptomatic route carries cases who will be diagnosed eventually, while the asymptomatic route contains cases who will never be diagnosed and will recover by themselves. In the proposed vSIADR model, the asymptomatic, the pre-symptomatic and yet to be diagnosed, and diagnosed are all contagious, which reflects the COVID-19 clinical reality that majority of secondary infections are made before being diagnosed [13] and the existence of asymptomatic infections [14]. Frequentist estimates to the time-varying infection, diagnosis and removal rates, and the effective reproduction number *R*_*t*_ are constructed by imputing conditional Poisson likelihood scores. Based on the estimated *R*_*t*_, we construct the counter-factual *R*_*t*_ under different policy scenarios, and compare the observed epidemic counts with those under the counter-factual experiments, for instance, China and Korea’s policies, the earlier or delayed actions in the USA and the UK in the early stage of the epidemic, and the strengthened or relaxed stringency measures for the fall-winter epidemics.

**Figure 1.**
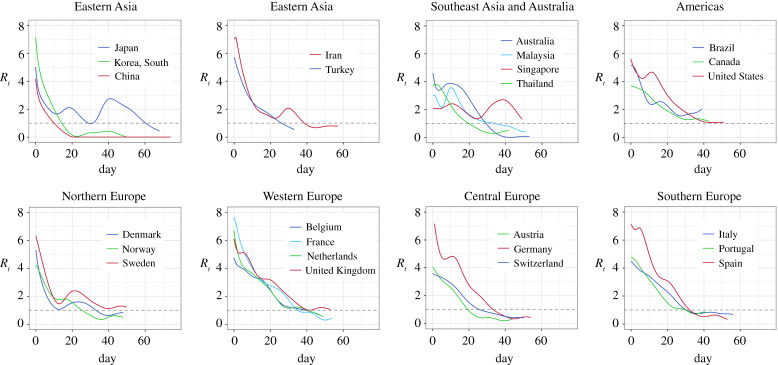
The estimated effective reproduction number *R*_*t*_ curves of 25 countries from their dates of community transmission (DCT, Day 0) to 20 April 2020 under the pre-symptomatic rate *θ* = 0.8. The dashed line represents the critical threshold level 1. (Online version in colour.)

Governments of the nations have implemented a range of counter measures to control the COVID-19 epidemics. Our study finds (electronic supplementary material, figure S1) significant negative correlations between the government imposed stringency scores, composed by Oxford Covonavirus Government Response Tracker (OxCGRT) [15], and the one to three weeks delayed effective reproduction numbers *R*_*t*_ from each country’s date of community transmission (DCT) to 31 December 2020. In particular, the average two weeks lag correlations was −0.7408 (SE: 0.024), indicating the overall reducing effect of the stringency measures on the *R*_*t*_. If we focus on the first wave of the epidemics, the time to reach the epidemic turning point was much influenced by the policy implemented within the first two weeks since the DCT with a negative correlation of −0.57 (*p*-value 0.002), which implies the effects of the stringency measures to shorten the time to the turning point of the epidemic. China (excluding Hubei Province) and Korea are found to be the most effective in bringing down the reproduction of COVID-19 in the first four weeks of community infections (table 1 and figure 1), and took the shortest time to reach the epidemic turning point in the first wave of the epidemics. The benefits of acting early with meaningful enforcement in reducing both the infection size and total deaths are demonstrated by counter-factual calculations under the Korea and China scenarios. Figure 5 shows reductions of 1.83 million (1.88 millions) confirmed cases and 139 321 (142 645) deaths among the other 23 countries would have been made if Korea (China)’s daily reduction percentage in the infection rates had adopted from Day 8 of each country’s start of community infection to 20 April, while maintaining each country’s removal and diagnostic rates. These numbers mount to 89% (91%) and 86% (88%) of the total infected cases and deaths of the 23 countries on 20 April 2020, respectively.

**Table 1.** Weekly averages of the estimated reproduction numbers *R*_*t*_ (W1-W4) of 25 countries over the four weeks from their respective start date of community transmission (DCT) under the pre-symptomatic rate *θ* = 0.8. Countries are ranked based on the average *R*_*t*_ over the four weeks (4W-Ave). China refers to the provinces excluding Hubei province. The 95% confidence intervals for *R*_0_ are available in table S4 in electronic supplementary material.

	country	DCT	*R*_0_	W1	W2	W3	W4	4W-Ave
1	China	01-23	4.17	2.18	0.90	0.16	0.01	0.81
2	Korea	02-17	7.14	4.05	1.80	0.41	0.14	1.60
3	Denmark	03-03	5.30	2.85	1.22	1.40	1.56	1.76
4	Thailand	03-09	3.57	3.46	2.10	1.16	0.61	1.83
5	Austria	03-07	4.07	3.33	2.40	1.25	0.49	1.87
6	Singapore	03-02	2.18	2.16	2.36	1.81	1.43	1.94
7	Norway	03-03	4.24	3.18	1.89	1.69	1.05	1.95
8	Japan	02-12	5.04	2.71	1.75	2.03	1.43	1.98
9	Malaysia	02-29	3.05	2.54	3.24	1.92	1.28	2.24
10	Switzerland	03-01	3.62	3.36	2.78	1.80	1.07	2.25
11	Turkey	03-18	5.72	4.12	2.42	1.75	1.10	2.35
12	Canada	03-08	3.68	3.50	2.83	2.08	1.53	2.48
13	Portugal	03-09	4.69	4.21	2.99	1.82	1.15	2.54
14	Sweden	03-01	6.46	4.31	1.78	2.11	2.19	2.60
15	Iran	02-23	6.94	5.20	2.37	1.58	1.58	2.68
16	Brazil	03-12	5.16	4.26	2.49	2.41	1.70	2.72
17	Italy	02-24	4.25	3.89	3.28	2.51	1.61	2.82
18	Belgium	03-04	4.76	4.04	3.41	2.78	1.63	2.96
19	Australia	02-27	4.62	3.58	3.82	3.14	1.75	3.07
20	Holland	03-02	6.70	4.56	3.51	2.68	1.58	3.08
21	USA	02-29	5.62	4.54	4.52	3.46	2.31	3.71
22	UK	02-27	6.12	5.14	3.95	3.21	2.61	3.73
23	France	02-26	7.66	5.81	3.86	2.89	2.41	3.74
24	Germany	02-26	8.36	5.39	4.68	3.28	2.09	3.86
25	Spain	02-27	7.14	6.71	4.59	3.19	2.25	4.19
	Ave		5.25	3.96	2.84	2.10	1.46	2.59
	SE		0.31	0.22	0.21	0.17	0.13	0.16

Our study shows that taking effective control measures can significantly impact the sizes of infections and deaths without having to mimic Korea and China’s experiences in the first wave of the epidemics. For the USA and the UK, if policy interventions had been made to ensure the declines in the *R*_*t*_ from 13 March for the USA [16] and 20 March for the UK [17] happen 5 days earlier (delayed), the USA would have reduced (increased) the cases and the deaths by 80 and 78% (384 and 315%), and the UK by 28 and 28% (42 and 37%), respectively, relative to the observed statistics on 20 April (figure 6). The USA and the UK experiments also inform the role played by the diagnosis rate *α* that regulates the speed of movement from the infected state to the diagnosed state. In particular, if the UK’s low diagnosis rate (0.1) had been applied to the USA (0.17), both the infection cases and the deaths would have been increased by 894 and 527% under the 5-day delayed setting.

To provide insights on the policy effects on the winter epidemic situation without vaccines, we conduct projections under different strength of control measures with the established linear mixed-effect model. It shows that any relaxation of the stringency measures from the current level would lead to significant increases of infection cases and deaths by the end of February 2021. The projections reveal that, compared to the scenario of maintaining the policy scores and NO_2_ levels as those on 31 December 2020, returning to the most strict policy measures would make confirmed cases decreased by 25.9% in average, and deaths reduced by 17.4% by 28 February 2021. If each country lowers its policy scores to half of its maximum policy levels and increases their NO_2_ to be twice of their minimums seen in 2020, the confirmed cases would increase by 833.2% in average, and six countries’ confirmed cases would increase more than 10 times. In addition, projected deaths would increase by 477.2% in average. This means that the levels of the counter measures should not be relaxed from the current level, and it is highly recommended to return at least to their maximum levels for some countries as specified in §6.

## 2. Data and methods

### (a) Data

We consider 25 countries in our study as listed in table 1, which had experienced COVID-19 with at least four weeks of established community infections on 20 April 2020. The daily records of infected, dead and recovered patients are obtained from Johns Hopkins University Center for Systems Science and Engineering [2] and WHO [3] for the 25 countries, supplemented by statistics from these nations’ health ministry and Dingxiangyuan Pneumonia website [4] for China’s data. The population sizes are from the United Nation [20]. In our study, data in China only contains those from the mainland provinces without Hubei province where Wuhan is the capital city due to incomplete observations at the start of the epidemic. The daily new cases, deaths and recoveries are smoothed to remove measurement errors and reporting delay. The smoothing procedure is outlined in the electronic supplementary material.

There are 11 countries which under-reported their daily recovery counts (Belgium, Canada, France, Italy, Netherlands, Norway, Portugal, Spain, Sweden, the UK and the USA), as shown by the much under-estimated recovery rates in electronic supplementary material, figure S2. We use 14 days as the average recover time from diagnosis, as suggested by WHO and supported clinically by [13], to impute the number of recovered cases for the 11 countries. For the other 14 countries, we use their reported data in the analysis.

Data on the 25 countries’ COVID-19 containment measures are collected in Oxford Coronavirus Government Response Tracker (OxCGRT) project [15], which collects publicly available information on 18 variables and aggregates them into sub-categories. In order to study the impacts of different policy actions, we use the stringency index, the economical support index and recalculate a health index without cross-combining them to form a composition score. Stringency index measures the strictness of government’s containment policy, such as restriction on gathering, suspension of schools and lock-down. The economic index measures a government’s economic support (cash support and debt relief), while the health index gauges on the government’s health-related actions including testing and contact tracing. We use the diagnosis testing scores from OxCGRT to demonstrate countries’ responses at the beginning of the pandemic in figure 3. We also construct an index based on daily NO_2_ concentrations using data provided by aqicn.org, which reflect the level of road traffics and hence the extent of the home isolation. Specifically, daily city-level NO_2_ concentrations is the median of observations from multiple monitoring stations in the city. The NO_2_ index is the ratio of the smoothed 2020 daily levels over the corresponding levels in 2019. NO_2_ index is not available for Singapore and Malaysia due to a lack of access to the data. Electronic supplementary material, figure S3 displays these scores for each country from 1 January 2020 to 16 January 2021.

### (b) Start dates and study period

The start date for established community transmission of a country is determined by considering the time when local infection emerged, the start date provided by the WHO [3] and the estimated infection rate under the proposed vSIADR model. It is set as the first local maximum of the estimated infection rate after the WHO date.

The study period for policy evaluations is from the start date of each country up to April 20 for the first wave of the pandemic, which is then extended to 31 December 2020 for analyses on later waves of COVID-19 reproduction and control measures for the 24 countries without China. The latter is due to China’s zero-tolerance policy which made any locally transmitted cases sporadic since April 2020 and the data uninteresting from a modelling point of view. Projections under different government policy counter-measure scenarios are made to 28 February 2021 based on data up to 31 December 2020.

### (c) Varying coefficient susceptible-infected-asymptomatic-diagnosed-removed model

Let *S*(*t*), *I*_*a*_(*t*), *I*_*p*_(*t*), *D*(*t*), *R*_*a*_(*t*), *R*_*r*_(*t*) and *R*_*d*_(*t*) be the counts of the susceptible, infected but asymptomatic, infected and pre-symptomatic, diagnosed, recovered from asymptomatic, recovered from diagnosed and dead people in a country at day *t*, respectively. Let *R*(*t*) be the sum of the recovered *R*_*r*_(*t*) and death *R*_*d*_(*t*), which is the number of removed from *D*(*t*) at time *t*. Let *N*(*t*) = *D*(*t*) + *R*(*t*) and Δ*N*(*t*) = *N*(*t* + 1) − *N*(*t*) be the accumulative and daily increment of confirmed cases at time *t*.

We propose a varying coefficient susceptible-infected-asymptomatic-diagnosed-removed (vSIADR) model. It extends the conventional SIR [11] model in four aspects: (i) separating the classical *I* state into the asymptomatic state *I*_*a*_ and the pre-symptomatic *I*_*p*_ state before being diagnosed, where only the diagnosed cases are observable; (ii) allowing infections in both the *I* states (*I*_*a*_ and *I*_*p*_) and the *D* state, where *I*_*a*_ and *I*_*p*_ are not observable; (iii) having asymptomatic cases as a new pathway from the *I* state in additional to the one for the pre-symptomatic cases; and (iv) allowing time-varying coefficients including the infection and removal rates. The first three aspects of the vSIADR model better capture the COVID-19 epidemics as most infections are made before being clinically diagnosed, and there are substantial asymptomatic cases which are never diagnosed and yet contagious [19,21,22]. Indeed, both SIR and SEIR [23] models assume the infected *I* state (after being diagnosed) is the only infectious state among the compartments. However, in reality, the infected after being diagnosed will be largely quarantined at home or hospitals and would have reduced contact rates and be less infectious.

The vSIADR model has the following ordinary differential equations (ODEs) which specify the conditional means of the *t* + 1 daily increments given the counts (*S*(*t*), *I*_*a*_(*t*), *I*_*p*_(*t*), *D*(*t*), *R*_*r*_(*t*), *R*_*d*_(*t*)) at time *t*:

2.1 $$\begin{array}{rl} & \frac{\text{d}S(t)}{\text{d}t}=-\{\beta_{t}^{{\text{I}}_{a}}I_{a}(t)+\beta_{t}^{{\text{I}}_{p}}I_{p}(t)+\beta_{t}^{\text{D}}D(t)\}\frac{S(t)}{M},\\ & \frac{\text{d}I_{a}(t)}{\text{d}t}=(1-\theta )\{\beta_{t}^{{\text{I}}_{a}}I_{a}(t)+\beta_{t}^{{\text{I}}_{p}}I_{p}(t)+\beta_{t}^{\text{D}}D(t)\}\frac{S(t)}{M}-\gamma_{r,t}I_{a}(t),\\ & \frac{\text{d}I_{p}(t)}{\text{d}t}=\theta \{\beta_{t}^{{\text{I}}_{a}}I_{a}(t)+\beta_{t}^{{\text{I}}_{p}}I_{p}(t)+\beta_{t}^{\text{D}}D(t)\}\frac{S(t)}{M}-\alpha I_{p}(t),\\ & \frac{\text{d}D(t)}{\text{d}t}=\alpha I_{p}(t)-\gamma_{t}D(t),\\ & \frac{\text{d}R_{a}(t)}{\text{d}t}=\gamma_{r,t}I_{a}(t)\;\\ \;\text{and}\; & \frac{\text{d}R_{r}(t)}{\text{d}t}=\gamma_{r,t}D(t)\;\text{and}\;\frac{\text{d}R_{d}(t)}{\text{d}t}=\gamma_{d,t}D(t), \end{array}\}$$

where $\beta_{t}^{{\text{I}}_{a}}$, $\beta_{t}^{{\text{I}}_{p}}$, $\beta_{t}^{\text{D}}$ are time-varying infection rates for the never diagnosed asymptomatic infections in *I*_*a*_, not yet diagnosed infection in *I*_*p*_ and the diagnosed infections in *D*, *θ* ∈ (0, 1) is the proportion of pre-symptomatic cases who will develop symptom and be diagnosed in the daily increase of infected cases, *γ*_*r*,*t*_ and *γ*_*d*,*t*_ are the time-varying recovery and death rates, respectively, *γ*_*t*_ = *γ*_*d*,*t*_ + *γ*_*r*,*t*_ is the overall symptomatic removal rate and *M* is the population size. Under Model (2.1), 1 − *θ* proportion of daily newly infected cases would become asymptomatic infections. The proportion *θ* and the diagnostic rate *α* are constants in (2.1), while extension to make them time varying can be made as there might be more infected cases not diagnosed at the beginning of the epidemics [19,22]. The selection and estimation of those parameters are discussed in the next subsection. Each equation in (2.1) may be divided by the population size *M* on both sides. In this sense, (*S*(*t*), *I*_*a*_(*t*), *I*_*p*_(*t*), *D*(*t*), *R*_*a*_(*t*), *R*_*r*_(*t*), *R*_*d*_(*t*)) become the proportions of the compartments such that their summation equals to 1.

A key difference between the SEIR and vSIADR models is that the latter does not *have* the non-infectious exposed E-state as the I state is already unobservable in vSIADR. We choose to hide the E-state in our model to avoid having two latent states in the pre-symptomatic pathway as otherwise it would create much disparity between model and data, and difficulties with estimation.

The time-varying coefficients in the model reflect the changing aspects of the COVID-19 pandemic in terms of the transmission and removal processes as well as the policy interventions implemented by various governments to contain the spread of the virus. The varying infection rates reflect the varying degrees of the containment measures taken by each country and individual behaviour changes (social distancing and face mask wearing) as well as the varying infectiousness of the virus over time. The varying removal rate accounts for varying medical conditions and improvement of treatments. For the infection rates, *although* the policies of a country can be fixed over a period of time, individual responses and behaviours to the policy measures may differ which can lead to varying dynamics in the spread of the disease. In any case, constant coefficients are special cases of the varying coefficient setting, and would be reflected in the empirical estimates by the local kernel regression outlined in §2d.

Let Δ*S*(*t*) = *S*(*t* + 1) − *S*(*t*), Δ*I*_*a*_(*t*) = *I*_*a*_(*t* + 1) − *I*_*a*_(*t*), Δ*I*_*p*_(*t*) = *I*_*p*_(*t* + 1) − *I*_*p*_(*t*), Δ*D*(*t*) = *D*(*t* + 1) − *D*(*t*), Δ*R*_*a*_(*t*) = *R*_*a*_(*t* + 1) − *R*_*a*_(*t*), Δ*R*_*r*_(*t*) = *R*_*r*_(*t* + 1) − *R*_*r*_(*t*) and Δ*R*_*d*_(*t*) = *R*_*d*_(*t* + 1) − *R*_*d*_(*t*) be the daily increments of the state variables, respectively. The stochastic version of the vSIADR model is constructed so that the daily increments follow the conditional Poisson processes with the conditional means specified by the daily discretized version of (2.1):

2.2 $$\begin{array}{rl} & -\Delta S(t)∼\text{Poisson}\{(\beta_{t}^{{\text{I}}_{a}}I_{a}(t)+\beta_{t}^{{\text{I}}_{p}}I_{p}(t)+\beta_{t}^{\text{D}}D(t))S(t)/M\},\;\Delta N(t)∼\text{Poisson}\{\alpha I_{p}(t)\},\\ & \Delta R_{a}(t)∼\text{Poisson}\{\gamma_{r,t}I_{a}(t)\},\;\Delta R_{r}(t)∼\text{Poisson}\{\gamma_{r,t}D(t)\},\;\Delta R_{d}(t)∼\text{Poisson}\{\gamma_{d,t}D(t)\} \end{array}$$

with initial values {*I*_*a*_(0), *I*_*p*_(0), *D*(0), *R*_*r*_(0), *R*_*d*_(0)} and the relationship $\Delta I_{a}(t)=\text{Binomial}(-\Delta S(t),1-\theta )-\Delta R_{a}(t)$, Δ*I*_*p*_(*t*) = −Δ*S*(*t*) − Δ*I*_*a*_(*t*) − Δ*R*_*a*_(*t*) − Δ*N*(*t*) and Δ*D*(*t*) = Δ*N*(*t*) − Δ*R*_*r*_(*t*) − Δ*R*_*d*_(*t*). The Poisson model can be weakened to be semi-parametric as the estimation can be made based on the conditional mean specification in (2.2).

The proposed model assumes that the depletion of the susceptible *S*(*t*) is made by the infected in the asymptomatic compartment *I*_*a*_, the pre-symptomatic but yet to be diagnosed cases in the *I*_*p*_ compartment and the confirmed (diagnosed) cases in the *D* compartment. The transmission rate of asymptomatic cases is lower than that of the pre-symptomatic cases [24,25] as supported by a range of clinical data. Focused on 455 contacts exposed to the asymptomatic COVID-19 virus carriers, [24] found no severe acute respiratory syndrome coronavirus 2 (SARS-CoV-2) infections was detected in all these contacts in the nucleic acid tests, and concluded that the infectivity of asymptomatic SARS-CoV-2 carriers might be weak. In a related work, [25] analysed a set of COVID-19 contact tracing surveillance data which included 161 symptomatic cases and 30 asymptomatic cases in Ningbo, China from 20 January to 6 March 2020. Using a SEIR modelling framework, they concluded that the relative transmissibility of asymptomatic case was significantly lower than that of the symptomatic cases. Besides, the contact rate of the cases after diagnosis is much reduced due to self-quarantine or hospitalization. Thus, the pre-symptomatic cases before being diagnosed are the most contagious group. We set $\beta_{t}^{{\text{I}}_{a}}=\beta_{t}^{\text{D}}=\beta_{t}^{{\text{I}}_{p}}/r$ for a constant *r* > 1 to reflect the reality of COVID-19.

The effective reproduction number under the vSIADR model, whose derivation is given in the electronic supplementary material, is

2.3 $$R_{t}=\theta ({\tilde{\beta }}_{t}^{\text{D}}/\gamma_{t}+{\tilde{\beta }}_{t}^{{\text{I}}_{p}}/\alpha )+(1-\theta ){\tilde{\beta }}_{t}^{{\text{I}}_{a}}/\gamma_{r,t},$$

where ${\tilde{\beta }}_{t}^{\text{D}}=\beta_{t}^{\text{D}}S(t)/M$, ${\tilde{\beta }}_{t}^{{\text{I}}_{a}}=\beta_{t}^{{\text{I}}_{a}}S(t)/M$ and ${\tilde{\beta }}_{t}^{{\text{I}}_{p}}=\beta_{t}^{{\text{I}}_{p}}S(t)/M$ are the discounted infection rates by the susceptible rate *S*(*t*).

We are aware of the fact that the above model framework is only an approximation to the reality and is subject to the specification errors as implicated by the empirical results in [26,27]. The varying coefficient aspect of the model reduces the potential risk of mis-specification. In the proposed estimation, we only use the mean information to formulate estimators without exploiting the mean-variance relationship under the Poisson assumption, which reduces the consequences of potential model mis-specification and increases the robustness of the proposed method.

### (d) Estimation

The parameter estimation is made in a two-step procedure. We first estimate ${\tilde{\beta }}_{t}^{{\text{I}}_{p}}$, *γ*_*d*,*t*_ and *γ*_*r*,*t*_ under the specification ${\tilde{\beta }}_{t}^{{\text{I}}_{a}}={\tilde{\beta }}_{t}^{\text{D}}={\tilde{\beta }}_{t}^{{\text{I}}_{p}}/r$ for a given *θ* ∈ (0, 1), *r* > 1 and the diagnosis rate *α*. Then, in the second stage, we estimate *α* and *r* based on a cross-validation measure under a chosen *θ*.

As the asymptomatic cases are latent, *θ* which regulates the distribution of infected to be symptomatic and asymptomatic can not be identified from the standard epidemiological statistics, and its value has to be assigned based on results from other studies. There are a diverse range of values for *θ* among existing studies including [19,22] which reported rather small *θ* values in the initial stage of the epidemics. [28] surveyed 79 datasets in published studies up to 10 June 2020, and found most of the asymptomatic cases become symptomatic as the time progresses. They reported the frequencies of symptomatic and asymptotic COVID-19 cases, and found 20% (CI: 17–25%) of COVID-19 infections remained asymptomatic among the 79 studies. The mega-analysis of [28] lends support for *θ* = 0.8, which is used as the baseline value in our analysis. As raised by a referee, there were under-reporting of the pre-symptomatic cases in some countries at the start of the epidemics, which had *aggravated* the situation. To consider the uncertainty with the underlying asymptomatic rate, two additional values of *θ* = 0.6 and 0.4 are also employed, and the results under these two values of *θ* are reported in electronic supplementary material, figure S4, which shows that the estimated *R*_*t*_ is robust with respect to *θ*.

Another challenge in the estimation for both the conventional SEIR model and the proposed vSIADR model is due to the latent states. The Bayesian method has been a commonly used estimation approach for the SEIR model [29–32]. Here, we present a frequentist approach by first imputing the latent *I*_*a*_(*t*) and *I*_*p*_(*t*) in building the estimating equations. Under the assumption that the conditional mean of the daily increments follows the specification (2.1), the following approximation can be entertained

$$\Delta I_{p}(t)\approx (\theta {\tilde{\beta }}_{t}^{{\text{I}}_{p}}-\alpha )I_{p}(t)+\theta {\tilde{\beta }}_{t}^{{\text{I}}_{p}}\{D(t)+I_{a}(t)\}/r\;\text{and}\;\Delta N(t)\approx \alpha I_{p}(t).$$

Thus, for a given *θ* and a given pair of *α* and *r*, we can impute *I*_*p*_(*t*) by ${\hat{I}}_{p}(t)=\Delta N(t)/\alpha$, and impute *I*_*a*_(*t* + 1) sequentially by

$$\frac{1-\theta }{\theta }\{{\hat{I}}_{p}(t+1)-(1-\alpha ){\hat{I}}_{p}(t)\}+(1-{\hat{\gamma }}_{r,t}){\hat{I}}_{a}(t)$$

based on the second equation of (2.1), where ${\hat{\gamma }}_{r,t}$ is the estimate of *γ*_*r*,*t*_ from (2.6) below. Using those imputed values for *I*_*a*_(*t*) and *I*_*p*_(*t*), we estimate ${\tilde{\beta }}_{t}^{{\text{I}}_{p}}$ via an estimating function

$${\hat{I}}_{p}(t+1)-{\hat{I}}_{p}(t)\approx (\theta {\tilde{\beta }}_{t}^{{\text{I}}_{p}}-\alpha ){\hat{I}}_{p}(t)+\theta {\tilde{\beta }}_{t}^{{\text{I}}_{p}}\{D(t)+{\hat{I}}_{a}(t)\}/r.$$

Specifically, let $Y_{t}={\hat{I}}_{p}(t+1)+(\alpha -1){\hat{I}}_{p}(t)$ and $X_{t}=\theta [\Delta N(t)/\alpha +\{D(t)+{\hat{I}}_{a}(t)\}/r]$. Around a time *t*, we regress *Y*_*t*_ on *X*_*t*_ by the locally weighted kernel regression estimator. The kernel estimation of ${\tilde{\beta }}^{{\text{I}}_{p}}(t)$, at the given *r* and *α*, minimizes the objective function

2.4 $$\sum_{i=1}^{T}{\left(Y_{i}-X_{i}\beta \right)}^{2}B\left(\frac{t-i}{h}\right)$$

with respect to *β*, where *B*( · ) is a boundary kernel modified from a usual symmetric kernel [33] and *h* is the temporal smoothing bandwidth. The use of the boundary kernel is to account for the boundary bias associated with the non-parametric regression near the ending time of the analysis; see the electronic supplementary material for the details. The kernel estimator for ${\tilde{\beta }}_{t}^{{\text{I}}_{p}}$ is

2.5 $${\hat{\tilde{\beta }}}_{t}^{{\text{I}}_{p}}=\frac{\sum_{i=1}^{T}X_{i}Y_{i}B\left((t-i)/h\right)}{\sum_{i=1}^{T}X_{i}^{2}B\left((t-i)/h\right)}.$$

Notice that the proposed estimator in (2.5) is based on the estimating equations of the means of the daily increments in the compartments. A conditional maximum-likelihood estimator (MLE) could be constructed using the distribution function of the difference between two Poisson random variables. However, this likelihood function depends on the infection rate parameters through the modified Bessel function of the first kind. The MLEs for the infection rate could be computationally unstable, specially for the local smoothing estimation where only observations in a local neighbourhood of *t* are used for estimating ${\tilde{\beta }}_{t}^{{\text{I}}_{p}}$. Although the proposed method is not a locally weighted MLE, it is robust to the distributional assumption of daily increments, and is computationally efficient and stable.

From the last two equation in (2.1), we have Δ*R*_*d*_(*t*) = *γ*_*d*,*t*_ *D*(*t*) and Δ*R*_*r*_(*t*) = *γ*_*r*,*t*_ *D*(*t*) in the conditional mean. Thus, we estimate *γ*_*d*,*t*_ and *γ*_*r*,*t*_ by regressing Δ*R*_*d*_(*t*) and Δ*R*_*r*_(*t*) on *D*(*t*), respectively, without intercept using the boundary kernel *B*(*t*) similar to (2.5):

2.6 $${\hat{\gamma }}_{d,t}=\frac{\sum_{i=1}^{T}D(i)\Delta R_{d}(i)B\left((t-i)/h_{d}\right)}{\sum_{i=1}^{T}D{\left(i\right)}^{2}B\left((t-i)/h_{d}\right)}\;\text{and}\;{\hat{\gamma }}_{r,t}=\frac{\sum_{i=1}^{T}D(i)\Delta R_{r}(i)B\left((t-i)/h_{r}\right)}{\sum_{i=1}^{T}D{\left(i\right)}^{2}B\left((t-i)/h_{r}\right)}.$$

It should be noted that the estimation of *γ*_*d*,*t*_ and *γ*_*r*,*t*_ are of one-step free of (*α*, *r*).

After obtaining estimation for the infection rate ${\tilde{\beta }}_{t}^{{\text{I}}_{p}}$ at a given (*α*, *r*), we conduct the second stage estimation for *α* and *r* based on a leave-one-out cross-validation criteria. Specifically, for each fixed (*α*, *r*), we estimate ${\tilde{\beta }}_{t}^{{\text{I}}_{p}}$ by the whole data except day *t*’s. Denote the leave-one-out version of (2.5) as ${\hat{\tilde{\beta }}}_{-t}^{{\text{I}}_{p}}(\alpha ,r)$ to highlight the role of (*α*, *r*). Let ${\hat{Y}}_{-t}(\alpha ,r)={\hat{\tilde{\beta }}}_{-t}^{{\text{I}}_{p}}(\alpha ,r)X_{t}$. Evaluate the fitting performance by comparing the relative difference between ${\hat{Y}}_{-t}(\alpha ,r)$ and *Y*_*t*_ by the following relative fitting criterion:

2.7 $$D(\alpha ,r)=\frac{1}{|T|}\sum_{t\in T}{\left|1-\frac{{\hat{Y}}_{-t}(\alpha ,r)}{Y_{t}}\right|}^{2},$$

and the selected (*α*, *r*) is $\left(\hat{\alpha },\hat{r})={\text{argmin}}_{\alpha \in \Lambda ,r>1}D(\alpha ,r\right)$, where T is a time period selected for evaluating the model fitting, and Λ is the plausible set of the diagnosis rate *α*. Based on the clinical information [13], we chose Λ = [0.1, 0.2] implying the average diagnosis time from 5 days to 10 days at the early stage of the epidemic. Finally, the estimate for the discounted infection rate ${\tilde{\beta }}_{t}^{{\text{I}}_{p}}$ is re-formulated according to (2.5) with the selected *α* and *r*. A parametric bootstrap procedure is proposed to conduct statistical inference associated with the various estimates; see the electronic supplementary material for details.

The estimated *α* are reported in table S1 of the electronic supplementary material. The estimated effective reproduction number curves *R*_*t*_ with respect to different *r* values are shown in electronic supplementary material, figure S5, which suggest both are not sensitive to the choice of *r*. Besides, the estimated effective reproduction number curves with different pre-symptomatic rate *θ* = 0.4, 0.6, 0.8 are shown in electronic supplementary material, figure S4, which suggest the robustness of the estimation of *R*_*t*_ with respect to the choice of pre-symptomatic rate *θ*.

### (e) Fitting and out-of-sample performance of vSIADR model

To gain information on the performance of the estimation procedure, we conducted simulation studies under three patterns of $\beta_{t}^{{\text{I}}_{p}}$, being constant, linearly increasing and linearly decreasing. Details of the simulation design are available in Section S**4** in the electronic supplementary material. Electronic supplementary material, figures S6-S7 display the simulation results for estimation of $\beta_{t}^{{\text{I}}_{p}}$, *γ*_*d*,*t*_ and *γ*_*r*,*t*_, while electronic supplementary material, table S2 shows those for the estimation of *α* and *r* via the cross-validation approach. They reveal general satisfactory performance of the estimation procedure. There was a noticeable bias and large variance in the first few days of the simulation due to small numbers of infected cases at the beginning of the epidemic, which disappeared quickly as more days were added to the simulation.

To gain further information on the fitting performance of the proposed vSIADR model, we display in electronic supplementary material, figure S8 the fitted values $\hat{\Delta N(t-1)}=\hat{N}(t)-N(t-1)$ under the fitted vSIADR model versus the observed *N*(*t*) − *N*(*t* − 1) for the 25 countries, where $\hat{\Delta N(t-1)}$ is calculated as $\hat{\alpha }({\hat{Y}}_{t-2}-(\hat{\alpha }-1){\hat{I}}_{p}(t-2))$ with ${\hat{Y}}_{t-2}={\hat{\tilde{\beta }}}_{t-2}^{{\text{I}}_{p}}X_{t-2}$ and ${\hat{I}}_{p}(t-2)=\Delta N(t-2)/\hat{\alpha }$. Electronic supplementary material, figure S8 shows that the fitted values were very close to the observed trajectories *N*(*t*) − *N*(*t* − 1) of the countries.

We also consider the out-of-sample validation for the vSIADR model by forecasting the sizes of new infections and deaths of a country. We use the prediction errors for the predicted new case numbers in the 7 days from 13 to 20 April, and the 14 days from 7 to 20 April, respectively, as the performance measures. The prediction of the epidemics is made by substituting the predicted **infection**, diagnosed, recovery and death rates into the vSIADR model (2.1) with initial values $\left\{\hat{S}(T),{\hat{I}}_{a}(T),{\hat{I}}_{p}(T),I(T),{\hat{R}}_{a}(T),R_{r}(T),R_{d}(T)\right\}$ at the current time *T*, for instance 12 April or 6 April for the two prediction exercises, respectively, where $\hat{S}(T)=M-{\hat{I}}_{a}(T)-{\hat{I}}_{p}(T)-{\hat{R}}_{a}(T)-N(T)$. If the estimated infection rates are decreasing in the last 7 days before *T*, we fit the reciprocal model $\beta_{t}^{{\text{I}}_{p}}=b/(t^{\eta }-a)+e_{t}$ with parameters *a*, *b* and *η* using the empirical estimates ${\hat{\beta }}_{t}^{{\text{I}}_{p}}$ from the last 7 days, and then project its values with the fitted parameters. If the estimated infection rates are not decreasing in the last 7 days, we predict $\beta_{t}^{{\text{I}}_{p}}$ using the average ${\hat{\beta }}_{t}^{{\text{I}}_{p}}$ over the last 7 days. The recovery and death rates are set as the averages of their empirical estimates in the immediate past 7 days, and the diagnosis rate *α* is the empirical estimate based on the data up to 6 April.

Electronic supplementary material, table S3 and figure S9 report the 7-day and the 14-day relative prediction errors for the 24 countries, where the errors were the ratio of the difference between the predict and the actual observed confirmed cases over the actual confirmed cases. It is seen that the relative errors for the number of new infections were generally small, averaged at 11.0 and 18.6% for the 7-day and 14-day predictions, respectively. Those results provided support for the vSIADR model and its estimation, and some assurance for its applications in the COVID-19 modelling and analyses.

### (f) Modelling *R*_*t*_ by policy scores

To gain knowledge on the effects of the COVID-19 counter measures on the reproduction number *R*_*t*_, we consider a linear mixed-effect model [18] which tries to explain the *R*_*t*_ movement in terms of the policies imposed by the governments. This is inspired by [19], which models the effective reproduction number *R*_*m*,*t*_ for the *m*th country at time *t* by scaling a baseline prior *R*_*m*,0_ with a linear combination of intervention indicators $\sum_{k}\alpha_{k}I_{k,m}(t)$ under the assumption that the effects of interventions are the same across all countries, where *I*_*k*,*m*_(*t*) equals 1 only if intervention policy *k* is imposed in country *m* at time *t*. In order to accommodate both common features shared by a group of countries and country-specific effects in the epidemiological processes, we consider using the linear mixed-effect model (LMM).

Let ${\hat{R}}_{m,t}$ be the estimated effective reproduction number for the *m*th country at time *t*, and *t* = 0 represent the DCT, the start date of community transmission. Let *s*_1,*m*_(*t*), *s*_2,*m*_(*t*) and *s*_3,*m*_(*t*) be the OxCGRT policy scores at time *t* corresponding to the country’s stringency measure, economics support and healthcare, respectively, and *s*_4,*m*_(*t*) be the NO_2_ score. To reduce noise in the policy scores, we conducted 3-day average of *s*_*k*,*m*_(*t*) and ${\hat{R}}_{m,t}$, and denote the corresponding average values as ${\bar{s}}_{k,m}(t)$ and ${\bar{R}}_{m,t}$. Due to the delayed policy effects as revealed in electronic supplementary material, figure S1, we consider the relationship between ${\bar{R}}_{m,t}$ and ${\bar{s}}_{k,m}(t-L)$, where the latter is the *L*-lagged values of the scores.

The LMM model for the policy effects on the effective reproduction number is

2.8 $${\bar{R}}_{m,t}={\bar{R}}_{m,0}\exp\left(b_{0,m}+\sum_{k=1}^{4}(\beta_{k}+b_{k,m}){\bar{s}}_{k,m}(t-L)+\epsilon_{m}(t)\right)$$

or equivalently,

2.9 $$\log\left(\frac{{\bar{R}}_{m,t}}{{\bar{R}}_{m,0}}\right)=\sum_{k=1}^{4}(\beta_{k}+b_{k,m})\Delta {\bar{s}}_{k,m}(t-L)+\epsilon_{m}(t),$$

where Δ*s*_*k*,*m*_(*t* − *L*) = *s*_*k*,*m*_(*t* − *L*) − *s*_*k*,*m*_( − *L*) is the change of the policy scores between (*t* − *L*)th day and the *L*th day before the DCT, *β*_*k*_ is a fixed coefficient representing the common effect of the *k*th score on the change of ${\bar{R}}_{m,t}$, and {*b*_*k*,*m*_} are the random effects of the *k*th score for each country, which are modelled as $b_{k,m}∼N(0,\sigma_{k}^{2})$ for *k* = 1, …, 4. The errors *ϵ*_*m*_(*t*) are assumed to be independently $N(0,\sigma_{\epsilon }^{2})$ distributed for each *t*. The lag value *L* = 12, representing the delay effect of the intervention policies, was chosen by minimizing the residual sum of square of the fitted model.

The advantages of the linear mixed-effect model (2.9) are in its incorporation of the group-common and individual-specific effects within a model. The fixed coefficient *β*_*k*_ represents the average effect of the *k*th policy on controlling the spread of COVID-19 across all countries. The random slopes {*b*_*k*,*m*_} reveal the country-wise specific effect for the *k*th score. If the variance $\sigma_{k}^{2}$ is not significantly non-zero, we may conclude that the *k*th policy had the same effect *β*_*k*_ for all countries in the group. Otherwise if the variance $\sigma_{k}^{2}$ is significantly non-zero, heterogeneous country-wise effects of the *k*th policy exist. For instance, a country with a negative (positive) *b*_*k*,*m*_ indicates a country-specific effect of the *k*th policy on deviating above (below) the common effect *β*_*k*_ on the effective reproduction of the epidemic. Hence, with the proposed LMM, we can estimate not only the commonly shared policy effect, but also conduct inference on the homogeneity of policy effects, and compare the heterogeneous effects among different countries.

Since the European and American countries have generally similar policy strategies and most of their epidemics are currently more severe than the countries in Asian and Australia, we divide the 24 countries into two groups: European and American countries as one group and Asian and Oceania countries as another. We fit the LMM (2.9) for the two groups separately by the *lmer* function in the *lme4 R* package, and predict the mixed effect {*b*_*k*,*m*_} by the best linear unbiased prediction (BLUP). Results are presented and discussed in §5.

## 3. Estimated effective reproduction numbers

**Figure 2.**
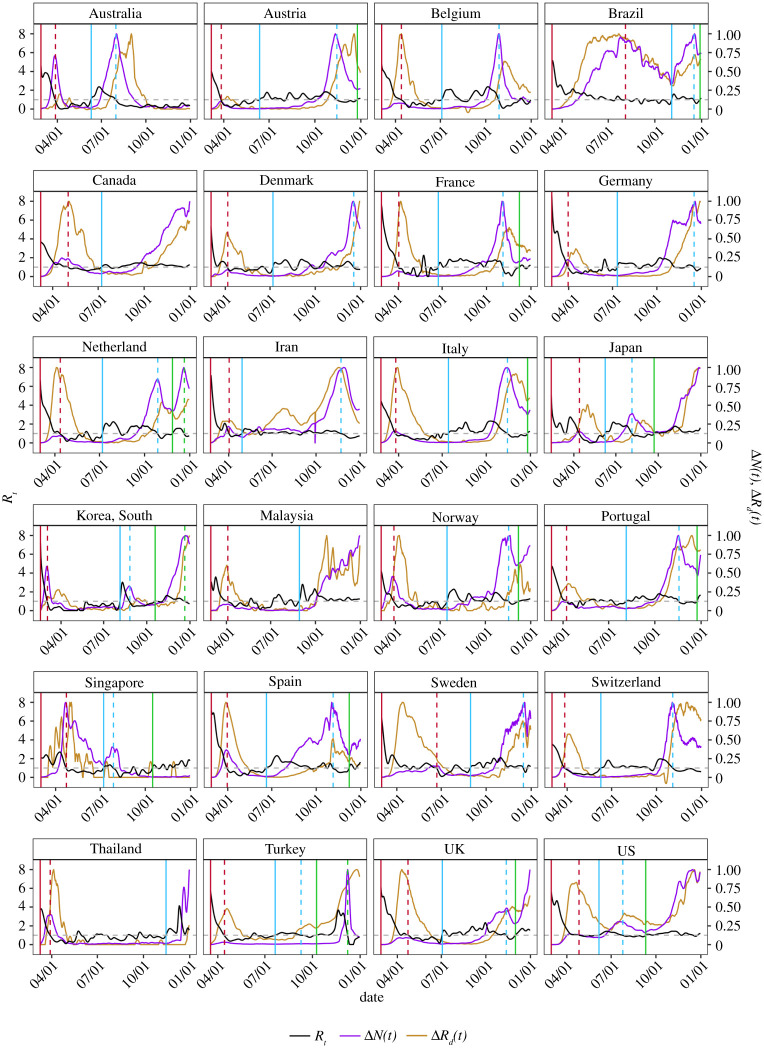
The estimated *R*_*t*_ curves (black), the daily new confirmed cases (purple) and the daily new death (yellow), both are scaled by the maximum level over the study period from the DCT to 31 December 2020 for the 24 countries (without China) under the pre-symptomatic rate *θ* = 0.8 with the start dates of the first (red), the second (blue) and the third (green) waves, and the dates of the turning point of the waves by dashed lines in the colour matching to that of the start date. The grey dashed line represents the critical threshold level 1.(Online version in colour.)

To evaluate community transmission and policy effects of COVID-19 across different countries, we first estimate the effective reproduction number *R*_*t*_ from the start date of each country’s local transmission under the vSIADR model using the estimation approach outlined in §2. Table 1 reports the estimated reproduction numbers *R*_*t*_ on the start date, and their weekly averages over the next four weeks. Figure 1 displays the *R*_*t*_ curves up to 20 April focusing on the first wave, while figure 2 shows the *R*_*t*_ up to 31 December for the first and second, and even the third waves of the epidemics in these countries. The average reproductive numbers on the start, which may be viewed as the basic reproduction number *R*_0_, was 5.25 (SE: 0.31) among the 25 countries with the lower and upper 25% quartiles being 4.24 and 6.39, respectively. The 14 European countries had higher *R*_0_, averaged at 5.65 (SE: 0.39). See table S4 in electronic supplementary material for the 95% confidence intervals of *R*_0_. As *R*_0_ is known to be subject to high volatility [34], one may look at the average *R*_*t*_ in the first week after the start of local transmission, which was 3.97 (SE: 0.22). Our estimate of *R*_0_ for non-Hubei China (4.78) is closer to that in [35] (*R*_0_ = 5.7 for Wuhan). Our estimated average *R*_0_ over the 25 countries was higher than the estimates 2–3 in [36,37] on Wuhan and 3.15 (CI: 3.04–3.26) in [8].

**Figure 3.**
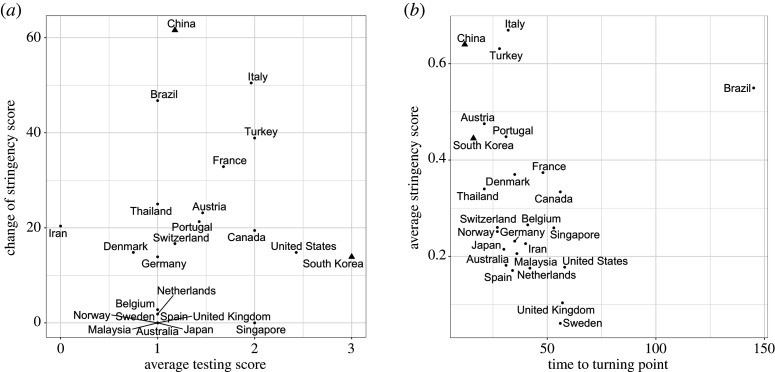
(*a*) Scatter plots of the average OxCGRT diagnosis testing scores in the first four weeks after the DCT and changes in the average OxCGRT stringency scores over a period from 5 days before to 5 days after the DCT; and (*b*) the average stringency scores in the first two weeks since the DCT of the countries versus their times to the epidemiological turning points in the first wave. The correlation in (*b*) after excluding the outlier Brazil was −0.557 (*p*-value 0.002). China and South Korea are marked with the triangle symbol.

Figure 1 shows that China and Korea’s *R*_*t*_ curves fell sharply below the other 23 countries’ over most of the four weeks after the start dates. Their rapid decline in the reproducing power was well reflected in table 1 as their average *R*_*t*_ over the four weeks were 0.81 and 1.6, respectively. By contrast, 17 countries had the four weeks average larger than 2, and seven of them more than 3. Not only that the absolute infectiousness of Korea and China were the lowest over the first four weeks, their cumulative percentages of declines were the most as shown in electronic supplementary material, table S5. The drastic decline in the reproduction of China was consistent with the studies [38,39]. Korea and China’s rapid declines in their *R*_*t*_ were due to their quick responses during the first four weeks of the epidemics, which included requiring face mask in public areas and reducing contact rates by sealing off outbreak areas and communities, promoting home isolation and enforcing quarantine for close contacts of diagnosed [8,40], which were effective measures in the early stage of the epidemic [5,12]. These were reflected in figure 3*a* which shows that China had the quickest increase in the stringency score within five days before and after the DCT, while Korea had the highest testing rates in the first four weeks. Indeed, Korea conducted active testing in its epidemic centre with more than half-million tests being carried out in the first month since the epidemic started [41,42].

By linking the estimated effective reproductive number *R*_*t*_ with respect to three categories of OxCGRT scores on the stringency, economic and healthcare-related counter measures to COVID-19, as well as an NO_2_-based index that reflects the level of road traffics and hence home isolation, we establish a linear mixed-effect regression model [18] for two groups of countries, (i) European and American countries and (ii) Asian and Oceania countries, from the start of epidemics to 31 December 2020, motivated by the work of [19]. The mixed-effect model allows common fixed effect parameters shared by all countries in a group while each country has individual random effect corresponding to different categories of counter measures. The statistical inference on the linear mixed-effect model (table 3) shows the stringency measures were the most significant in reducing the reproductive power of COVID-19 in all countries, and the economic and the NO_2_ indices were significant in the European-American group.

**Figure 4.**
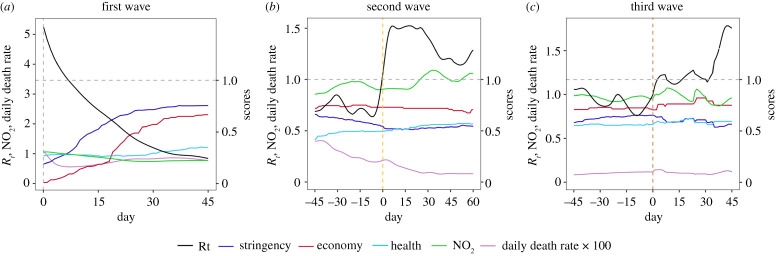
Average estimated *R*_*t*_ (black), NO_2_ index (green) and the average daily death rate *γ*_*d*,*t*_ multiplied by 100 (purple), in the left scale, over the countries which have experienced the first (*a*), second (*b*) and third (*c*) waves of the pandemic with the corresponding average relative OxCGRT scores (right scale) of stringency (blue), economy (red) and health (light blue) divided by their maximum value 100 from DCT to 45 days after the DCT in the first wave, 45 days before to 60 days after the start of the second wave and 45 days before and after the start of the third wave.(Online version in colour.)

Twenty four out of the 25 countries have experienced the second wave of the epidemics since 29 April 2020. Our analysis (figure 4) shows much lower level of counter measures in the three weeks leading to the start of the second wave, with the important stringency score decreased to 69% (SE: 0.03) of the maximum level sustained in countering the first wave epidemics. And quite alarmingly, after the start of the second wave, there was no immediate increase in the stringency measures. It appears that the governments’ only response to the second wave was in the healthcare area as reflected in the gradual increase in the health-related score in figure 4. The responses of most governments to the second and third wave were much slower and insufficient as compared with the first wave.

We identify the time points when the estimated *R*_*t*_ started to go above the critical threshold level 1 and stayed so over a period of time as the period of the first, second or the third wave of a country. Figure 2 shows a strong correspondence between *R*_*t*_ > 1 and the substantial increase of newly reported cases Δ*N*(*t*). Figure 2 also displays the estimated *R*_*t*_ curves with marked start and ending dates of the first, second and the third (if any) waves for each country. Table 2 provides the start dates and the lengths of each wave for the 25 countries. Among the 24 countries having had the second wave, Canada, Malaysia and Thailand were still in the middle of it without reaching the turning point (defined as the first time when *R*_*t*_ < 1). By 31 December, 14 countries have started the third wave and three of them have reached the turning points. The Kaplan-Meier estimate [43] for the average length of the second wave was 113 (SE 11.2), which was almost three times of the first wave (43, SE 5.9). The average gap time between the first and second waves was 92 days (SE: 8.4), and that between the second and third waves was 38 days (SE: 4.5). As shown in figure 2, although the average *R*_*t*_s in the second and third wave were not as high as those in the first wave, the daily new cases increased much more as the size of the infected population was much larger in the later waves. Thus, one should not think the relative low *R*_*t*_ in the second and third wave would suggest the *epidemics* in the second and third waves was less severe.

**Table 2.** Start date, length (in days) and the number of death between the start date and the turning point of the first, second and third waves in the 25 countries from their DCT to 31 December 2020. The average length of the second wave and their standard error estimated by the Kaplan–Meier method [43] are provided in parentheses. The columns headed by ‘Ratio’ report the ratios of the average daily death in the second (third) wave to that in the first wave. Empty cells mean the third wave has not started.

		first wave			second wave				third wave			
	country	DCT	length	death	start	length	death	ratio	start	length	death	ratio
1	Australia	02–27	30	14	06–10	51	99	4.16				
2	Austria	03–07	20	58	06–12	155	1071	2.38	12–25	6+	439	25.23
3	Belgium	03–04	40	3903	07–04	116	1399	0.12				
4	Brazil	03–12	145	95819	11–03	44	24331	0.84	12–29	2+	2268	1.72
5	Canada	03–08	55	3684	07–08	176+	6846	0.58				
6	Denmark	03–03	34	187	07–07	164	398	0.44				
7	France	02–26	36	5396	06–23	134	9005	0.45	12–08	23+	8308	2.41
8	Germany	02–26	34	775	07–11	159	15957	4.40				
9	Holland	03–02	41	2747	07–07	113	1114	0.15	11–27	24	1263	0.79
10	Iran	02–23	39	3031	04–29	207	38925	2.38				
11	Italy	02–24	31	8208	07–14	123	9699	0.30	12–26	5+	2539	1.92
12	Japan	02–12	61	156	06–08	58	108	0.73	09–22	100+	1773	6.93
13	Korea	02–17	15	28	08–05	21	11	0.28	10–19	63	275	2.34
14	Malaysia	02–29	35	53	08–29	124+	346	1.83				
15	Norway	03–03	26	25	07–15	125	45	0.37	12–07	24+	77	3.34
16	Portugal	03–09	30	380	08–05	105	1892	1.42	12–24	7+	493	5.56
17	Singapore	03–02	52	12	07–08	20	1	0.22	10–16	76+	1	0.06
18	Spain	02–27	33	10913	06–20	138	10044	0.22	12–08	23+	4191	0.55
19	Sweden	03–01	113	5346	08–30	108	1963	0.38				
20	Switzerland	03–01	26	231	06–09	147	537	0.41				
21	Thailand	03–09	20	6	11–15	46+	3	0.22				
22	Turkey	03–18	27	1402	07–21	50	1311	0.50	10–09	60	6592	2.12
23	UK	02–27	56	21855	07–03	132	10354	0.20	12–01	30+	14474	1.24
24	US	02–29	57	57281	06–06	49	34230	0.70	09–10	112+	154100	1.37
25	China	01–23	11	9								
	average		43			107+(113)		0.99		40+		3.97
	SE		5.9			10.7(11.2)		0.2		9.7		1.7
	total deaths		221 519			169 689				196 793		
	total confirmed cases		4 593 776			12 407 837				16 626 578		

Table 2 shows a substantial reduction in the death rate in the second wave in most countries relative to the first wave. Despite the overall average of the daily death ratios in the second wave to that in the first wave was close to 1 (98.7%, SE 25%), 18 of the 24 countries had the daily death ratio less than 0.85. The average daily death ratios was 68.8% (SE 15%) if we exclude Australia and Germany whose daily death ratios in the second wave were more than 4. Figure 2 also shows that the daily increase of death in the second wave was smaller than that in the first wave for most countries, especially for the European countries. But the death number started to climb up at the end of second wave for some countries due to the increase in the cases, while the numbers of death stayed at high level after the turning points due to the delay effect. We see a much higher daily death rate in 11 of the 14 countries having had the third wave. The average daily death ratios in the third wave relative to the first two waves in the 14 countries were very high, reaching 3.97 and 5.40, respectively, indicating a very grim situation.

Perhaps it was because of the relative low case numbers (hence low deaths) in majority of countries and a stringency fatigue, the second wave failed to arouse sufficient counter measures of the governments in most of the countries, which led to the prolonged duration of the second wave (average 113 days, SE 11.2) with three countries still not emerging from it on 31 December. The lack of governments’ response to the second wave was clearly shown in figure 4 which presents the average estimated *R*_*t*_ and the four average scores related to control measures in the 24 countries which had experienced the first two waves of epidemics. The figure demonstrates concordance in the changes of *R*_*t*_ and the policy scores, which confirms the sensitivity of the epidemics to the control measures. In particular, the decrease in *R*_*t*_ after the start of the first wave was highly correlated with the large increase in the stringency and economy scores. However, the very important stringency index was decreasing till the start of the second wave, indicating relaxed stringency measures before the start of the second wave. Only the health index increased in the first 45 days of the second wave, while the average stringency measures were flat at less than 55% of the maximum stringency strength seen in the first wave. This suggests the governments were reluctant to resort to the maximum stringency in the second wave. For the third wave, we observed that in the first 45 days of the third wave, the average stringency measures were more than 65% of the maximum stringency strength, but still largely flat, and the economy index increased from 70.31 to 82.14% of the maximum strength 25 days (in average) after the start of the third wave.

## 4. Scenario analysis and evaluation

Part of the COVID-19 control measures were designed to reduce the infection rates by limiting the contact probability. Thus, policy scenarios are made by altering the infection rates and hence the number *R*_*t*_ while keeping each country’s other epidemic parameters: the diagnose rate *α*, the recovery and death rates *γ*_*r*,*t*_ and the *γ*_*d*,*t*_ the same within the proposed vSIADR model while fixing the pre-symptomatic rate *θ* = 0.8. Epidemiological scenarios of Korea and China are generated for other countries in the early stage of the pandemic by applying the daily change percentages of the estimated infection rates of Korea and China from the eighth day since the starts of the community transmission. Similar designs are used for the UK and the US experiments for earlier and delayed intervention scenarios.

### (a) Epidemic projections under Korea and China’s scenarios

Given the effectiveness of Korea and China’s approaches in containing COVID-19 in the early stage of the epidemic, we generate scenarios for other countries that mimic Korea and China’s daily reduction percentages in the infection rates from the 8th day since the start of local transmission, while keeping their diagnosis, recovery and death rates intact. The generated numbers after Day 8 create scenarios for other countries (shown in electronic supplementary material, figure S10) where the control measures of Korea and China were implemented.

**Figure 5.**
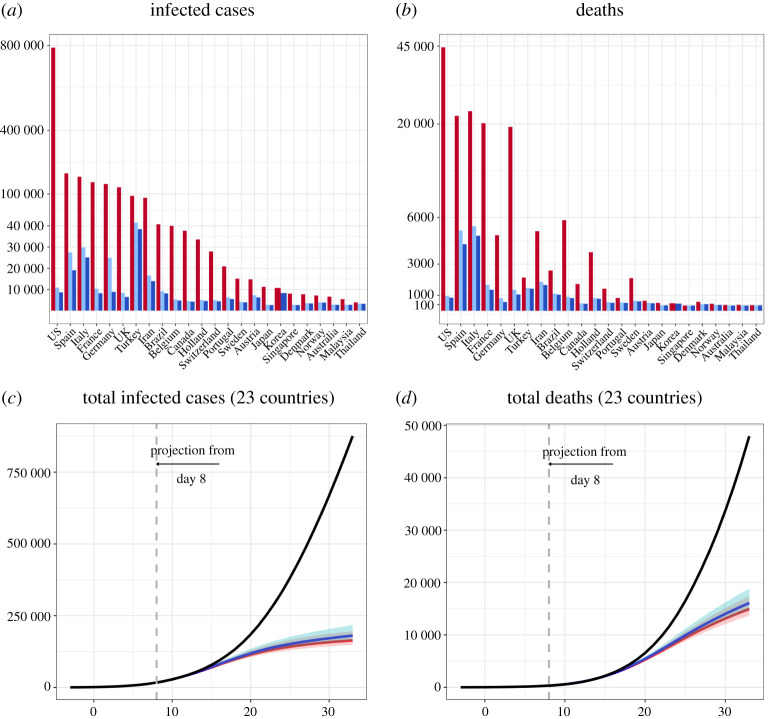
The observed numbers (red bar) of infected cases (*a*) and deaths (*b*) of the countries and the would-be ones under China (blue bar) and Korea (light blue bar)’s scenarios implemented from Day 8 of community transmission to 20 April; and the observed (black) total numbers of infected cases (*c*) and deaths (*d*) of the 23 countries excluding Korea and China and the would-be totals from Day 8 to Day 33 under China (red) and Korea (blue)’s scenarios. The pre-symptomatic rate *θ* is 0.8.(Online version in colour.)

Comparing to the actual cases observed up to 20 April, figure 5 shows that 1.83 (1.88) millions reductions in the confirmed cases and 139 321 (142 645) reduction in deaths for the 23 countries under the Korea’s (China’s) scenario, mounting to 89% (91%) and 86% (88%) reductions of the confirmed cases and deaths on average, respectively (more details in electronic supplementary material, table S6). The reductions in the cases and deaths would have been phenomenal for the USA, Japan, the UK and France, attaining more than 92% reductions in the confirmed cases and 86% reduction in deaths under Korea’s scenario, if control measures had been implemented earlier that would lead to the reductions in the infection rates from Day 8 of community transmission. And those under China’s scenario would be a few percentage points more.

### (b) Evaluation on the USA and the UK

**Figure 6.**
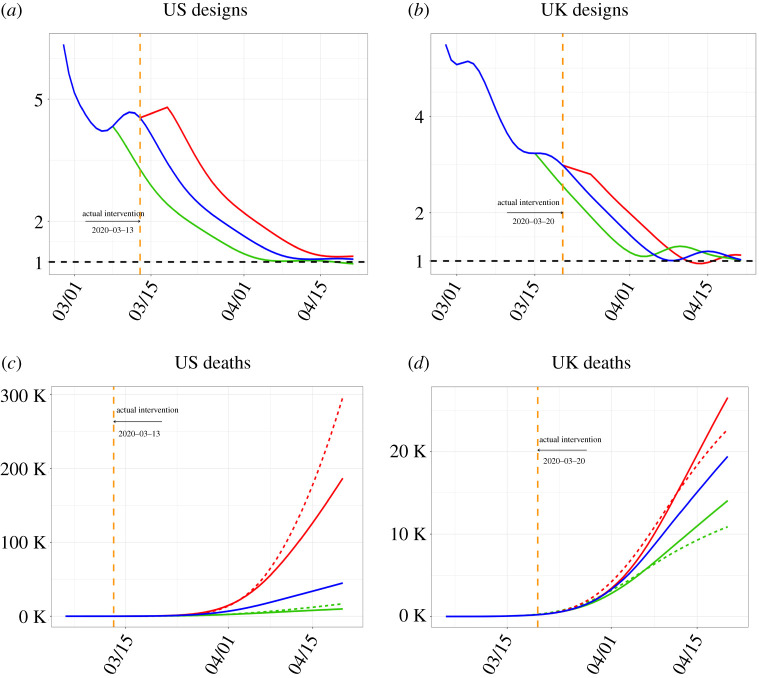
The effective reproduction number *R*_*t*_ curves (blue lines) and the *R*_*t*_ curves under the 5-day earlier (green lines) and the 5-day delayed (red lines) for the USA (*a*) and the UK (*b*), and the deaths (*c*,*d*) of the observed (blue) and those generated under the 5-day earlier (green) and 5-day delayed (red) experiments. Those dashed lines are deaths with the USA and the UK exchanging their diagnosis rate *α*: *α*_USA_ = 0.17 and *α*_UK_ = 0.1. The orange dashed lines mark the dates of the control measures of the two countries. Here, we use pre-symptomatic rate *θ* = 0.8. The results are up to 20th April.(Online version in colour.)

We consider two policy intervention experiments specific to the USA and the UK, which represent early and delayed implementation of counter-COVID-19 measures. 13 March and 20 March were the dates of firm policy measures by the USA and the UK governments, respectively, when the USA declared the national emergency and the UK started to close schools and public facilities. It appears from figure 6 that sustained declining trend of *R*_*t*_ was established after 13 March and 20 March for the USA and the UK, respectively. The experimental design of 5-day earlier intervention would mean the *R*_*t*_ curves start to decline from 8 March for the USA and 15 March for the UK at the actual daily declines rates from 13 and 20 March, respectively. The 5-day delayed experiments is to mimic later actions that would delay the decline in *R*_*t*_ from 13 and 20 March for 5 days to 18 and 25 March, respectively. Figure 6*a*,*b* display the actual *R*_*t*_ curves with the ones under the two designs.

The numbers of death cases under the two experiments are reported in figure 6*c*,*d*; see electronic supplementary material, table S7 for the specific numbers of death and total cases, including asymptomatic cases along with the observed statistics up to 20 April. Our result shows that by acting earlier both countries would have seen substantial reductions in the total number of infected cases and deaths: the cases and deaths in the USA would have been reduced by about 80 and 78% on 20 April, respectively; and the UK by 28 and 28%, respectively. By contrast, under the 5-day delayed postulations, the cases and deaths in the USA would have increased by 384 and 315%, and the UK by 42 and 37% on 20 April, respectively.

The above results indicate that the USA was more responsive to the intervention than the UK, as the earlier (delayed) intervention would reduce (increase) more infection cases and deaths than the UK. This can be explained in several aspects. First, the absolute decline of US’s *R*_*t*_ was 2.46, more than the UK’s 1.65 over the two weeks from 13 and 20 March, although the relative decline were comparable at 54 and 55%, respectively. Second, the US implemented the intervention relatively earlier as 13 March was the 14th day after the US’s start date while 20 March was the 24th days for the UK. Thus, the USA would have more time to amplify the effect of the intervention.

A less obvious reason for the differential sensitivity lies in the estimated diagnosed rates: 0.17 for the USA and 0.1 for the UK, implying the UK having longer time for diagnosing the infected than the USA. The larger diagnosis rate for the USA means quicker turn-over time from exposure to diagnosis, which would bring early peak time for the active infected cases *I*(*t*) and reduce the size of infections and hence the death number. Our result shows the numbers of cases for the USA with the UK’s diagnosis rate (0.10) would have amplified by more than 510% and the deaths by 212% on 20 April under the 5-day delay design. For the UK with the US’s higher diagnosis rate (0.17), there would have been a further 16% reduction in the number of total cases on 20 April under the 5-day early design, where the impacts on the death was slight. A high diagnosis rate is part of the Korea’s counter COVID-19 strategy.

## 5. Policy effects on *R*_*t*_

The countries have implemented a range of policies as counter measures to control the COVID-19 pandemic. Electronic supplementary material, figure S1 reports significant negative correlations between the OxCGRT scores with the 0-3 weeks delayed effective numbers *R*_*t*_ for all 25 countries from each country’s DCT to 31 December 2020. It shows that the correlation between the stringency score and the one or two (three) weeks lagged *R*_*t*_ being the highest in 18 (6) countries, confirming the natural delay effect of the policy. In particularly, the average one and two weeks lag correlation were −0.718 (SE: 0.031) and −0.7408 (SE: 0.024), respectively, among the 25 countries, indicating the overall effect of the control measures.

If we focused on the first wave of the epidemics, 24 out of the 25 countries (except Brazil) had reached the turning point of infection on 2 May 2020. Here the turning point is defined as the first day that *R*_*t*_ < 1 since the start of community infection and has stayed so for 7 consecutive days. The average time to the turning point for the 24 countries was 39.7 days (SE: 3.97). It took Brazil 145 days to reach the turning point, more than double the second longest of 58 days by the US as shown in figure 3*b*, which led us to treat Brazil as an outlier. Figure 3*b* also presents the scatter plot of the average OxCGRT stringency index within the first two weeks after DCT and the time to the turning points. The correlation between the average stringency score in the first two weeks since the DCT and the time to the turning point was −0.57 (*p*-value: 0.002) (excluding Brazil), indicating the overall effects of mounting the counter measures within the first two weeks of the epidemic for turning around the epidemics sooner.

In additional to the correlation analysis, we fit the LMM (2.9) for the European-American group and the Asian-Oceania group separately. China is not included in the Asian-Oceania group due to its rather short local transmission period and most of the infections since April were imported cases. Table 3 summarizes the estimation results for the fixed effects *β*_*k*_ and the variances $\sigma_{k}^{2}$ of the random effects, together with their significance of testing *β*_*k*_ = 0 and $\sigma_{k}^{2}=0$, and the BLUP for the random effects {*b*_*k*,*m*_} under the LMM for those two groups of countries. Electronic supplementary material, table S8 reports the coefficients of determination *R*^2^ for the countries as goodness-of-fit measures for their fitted curves as a function of the policy scores. The results show that the fitting performance of the proposed LMM was generally good with average *R*^2^ being 0.554 for European and American countries and 0.372 for Asian and Oceania countries, respectively. Lower levels of *R*^2^ were observed for Sweden (0.151), Denmark (0.190) and Iran (0.277), largely due to infrequent changes in the policy scores.

For the European-American group, the stringency, economic and mobility (NO_2_) scores were all significant at the 5% significance level, where stringency and economic were negative and the mobility index was positive, implying that a higher stringency level, cash assistance and debt reliefs offered by the countries were effective in during the epidemics. It also shows that more mobility (increased NO_2_) encouraged the increase of infection. The variance of the random effects for the four scores were all significantly not zero, implying there were individual country-specific effects for the four factors.

The NO_2_ data were not available for Singapore and Malaysia. We fitted the LMM with the NO_2_ index for the remaining countries in the Asian-Oceania group, which showed that the NO_2_ index was not significant. As a result, we did not consider NO_2_ in the model for the group. Table 3(b) shows that the fixed effect *β*_*k*_ and random effect variance $\sigma_{k}^{2}$ were significantly non-zero only for the stringency index among the three OxCGRT indices. The magnitude of the estimated *β*_1_ of the Asian-Oceania group was about twice of that of the European-American group. As *β*_1_ indicates the average reduction of log (*R*_*m*,*t*_) over all countries in the group for one unit increase in the stringency score, this shows that stringency policy in general was twice more effective for Asian and Oceania countries than that for the European and American countries. For the economic relief effects, since the absolute estimated *β*_2_ of the European-American group was larger than that of the Asian-Oceania group, while *β*_2_ was not significant for the Asian-Oceania group, the economic relief measures were more effective in the European and American countries.

**Table 3.** Estimates (multiplied by 10^2^) for the common policy effects and the standard deviation (*σ*) of the individual random effects together with their level of significance as reflected by the number of * under the linear mixed-effect model for (a) European and American countries and (b) Asian and Oceania countries using data from DCT to 31 December 2020, where *β*_1_, …, *β*_4_ represent coefficient of stringency, economics, healthcare and NO_2_ related index, respectively.

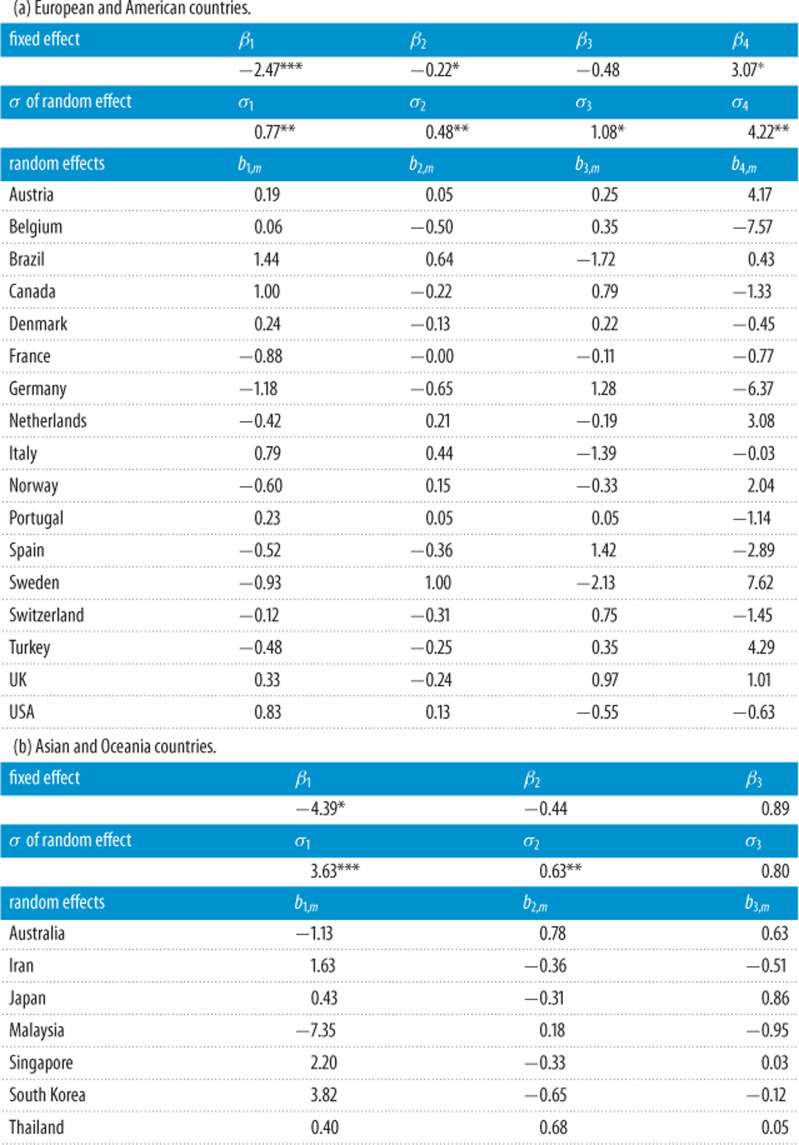

For country-specific effects of the stringency measures within the European-American group, it is noted that the five most negative estimated random effects *b*_1,*m*_ were Germany, Sweden, France, Spain and Turkey (in increasing order). This implies that these countries had the most effective stringency measures among the European-American group. While, the least effective countries are Brazil, Canada, the USA, Italy, the UK with highest estimated *b*_1,*m*_ in positive range, which also had the most and least effective economic relief measures, respectively, based on the ranking of the estimated *b*_2,*m*_.

## 6. Projection based on policy scenarios

Based on the vSIADR model and the LMM, we conduct projections from 31 December 2020 to 28 February 2021 under different settings of control measures to show how the epidemics would evolve under three different strategies of policies. The first scenario (Current Scenario) is to maintain the three policy scores as well as the NO_2_ levels of each country as those on 31 December 2020 for the target months of January and February 2021. The scenario represents a situation that the countries would maintain policy measures as they were on 31 December 2020. The second scenario (Maximum Scenario) assumes each country adopts the strongest policy intervention it has taken in the pandemic, meaning the policy scores at their respective maximum values and NO_2_ levels at the minimum throughout January and February 2021. Projections under this scenario show how the epidemics would evolve if the control measures are strengthened to their maximum levels again. The third scenario (50% Scenario) has the policy scores in January and February 2021 behalf of the historic maximums and twice of the minimum NO_2_ for each country. To gain information on the practical relevance of such projections, we conducted one-month projections under the three scenarios using data up to 15 December 2020 to predict the state variables on 15 January 2021 as displayed in electronic supplementary material, figures S11 and S12. It is shown that the errors in the projected deaths and cases standardized by population sizes were small under the scenario closest to the actual policy measure of the country during the projection period.

It is noted that there were some increases in the stringency and economic scores in the last two months of 2020 as more countries entered the more deadly third wave. The averages of the two scores for the 24 countries on 31 December were 83.7 and 90.2% of the historic maximums, respectively, up 15 and 4.7% from those on 30 October 2020. However, the average health score was down by 1.9% over the period.

For the Current Scenario, the projected *R*_*t*_ is chosen as its estimated value on 31 December 2020 according to the LMM (2.9). For the Maximum Scenario, we use a fraction of the estimated *R*_*t*_ on 31 December 2020 by a discount factor equal to the ratio of the fitted *R*_*t*_ under the strongest policy scores over the actual scores on 31 December 2020; and likewise for the 50% Scenario. Given that policy measures have delayed effect on *R*_*t*_, a two-week transitional period is set to linearly transform the *R*_*t*_ on 31 December 2020 to the projected value under a scenario on 13 January 2021, which is then kept fixed until 28 February 2021. We then solve the infection rates $\beta_{t}^{{\text{I}}_{p}}$ and $\beta_{t}^{{\text{I}}_{a}}=\beta_{t}^{\text{D}}=\beta_{t}^{{\text{I}}_{p}}/r$ from the expression of *R*_*t*_ in (2.3) with the pre-symptomatic rate *θ* = 0.8. Other epidemic parameters *γ*_*r*,*t*_, *γ*_*d*,*t*_, *α* and the infection ratio *r* of pre-symptomatic cases to asymptomatic cases were set as the estimated values based on the data up to 31 December 2020. We substitute those parameters into the conditional Poisson-vSIADR framework (2.2) to project the dynamic epidemiological state variables over the projected period. Projection intervals are constructed using the same procedure as the 95% confidence intervals of *R*_*t*_ (in the electronic supplementary material) via the LMM.

**Figure 7.**
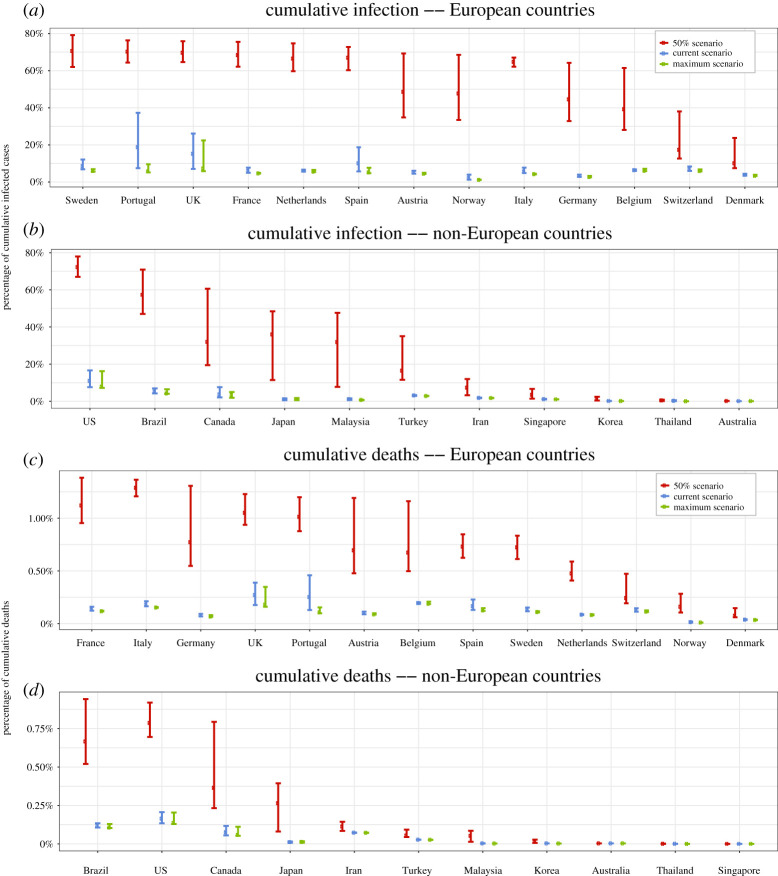
Projected relative cumulative infection and death cases and the 95% prediction intervals on 28 February 2021 for the thirteen European countries (*a*,*c*) and the 11 non-European countries (*b*,*d*) with the pre-symptomtic rate *θ* = 0.8 under the three policy scenarios: Current Scenario (blue) where the countries keep policy index measures as 31st December ; the Maximum Scenario (green) where each country adopts the strongest policy it has taken since the start of the epidemics; and the 50% Scenario (red) where the policy scores in projection period are half of the historic maximums for each country and also twice of the minimum NO_2_ for the European and America countries in the projection. Numerical results are in tables S9–S10 of the electronic supplementary material. The data used for the projections were up to 31 December 2020. (Online version in colour.)

The projected results under the scenarios are summarized in figures 7 for the ratio of cumulative confirmed cases and the number of death relative to the population total of respective country with the 95% projection intervals. The absolute numbers of the projected infections and deaths are reported in electronic supplementary material, figures S13 and S14, while the total number of infections (including asymptomatic cases) are reported in figure S15. Compared to the Current Scenario, the Maximum Scenario would have the confirmed cases decreased by 25.9% in average among the 24 countries by the end of February 2021, and eight countries decreased by more than 30%. The total infection size including asymptomatic cases would decrease by 31.4%, averaged over the 24 countries, with 11 countries decreased by more than 30%. Under the Maximum Scenario, for the three countries currently in the second wave and 10 of the 11 countries currently in the third wave, their *R*_*t*_ would drop below 1 by the end of February 2021 implying that the second or third wave of the epidemics could be finished by then. However, Japan, who is currently in the third wave, needs to apply 134% of the its past maximum levels of the control measures in order to attain the turning point before the end of February.

Under the 50% Scenario (half of the maximum policy scores and twice of the minimum NO_2_ level), the confirmed cases would increase by 833% in average, and for six countries (Japan, Malaysia, Norway, Germany, France and Italy), the confirmed cases would increase more than 10 times as compared to those under the Current Scenario. The total infections would increase more dramatically by 1066% in average among the 24 countries. One reason for the dramatic change is that with the half of the maximum policy scores, some countries whose current *R*_*t*_ levels were relatively low on 31 December 2020 would exceed 1, leading to exponential growth of the infections. Under this scenario, all countries which were in the second or third wave would remain so, as their *R*_*t*_ would be above 1 under the relaxed control measures.

On the number of death, compared to the Current Scenario, the average projected deaths would be reduced by 17.4% under the Maximum Scenario and increased by 477.2% under the 50% Scenario among the 24 countries. The relative less reduction in death under the Maximum Scenario was partly due to the difference between the score values on 31 December 2020 and the Maximum scores having become smaller. The projection results also reveal that for countries with low current infection cases such as Singapore and Australia, whose total infection size were under 500 at the end of 2020, it is comparatively safe to relax the policy to some extent as the projection results are not sensitive to the three scenarios. However, for countries like Japan, Sweden, the USA and France, if their policies were reduced to 50% of the maximum levels, the confirmed cases at the end of the February 2021 would increase over 550% of those under the Current Scenario, and deaths more than tripled. These are due to the four countries had at least one of the following characteristics on 31 December 2020: (i) high *R*_*t*_; (ii) excessive stock of infection, for instance the USA had over 1.9 millions infected cases; (iii) relative lower maximum level of stringency score than most countries. The maximum stringency scores of Japan and the Sweden were the first and third lowest among the 24 countries, respectively. It is expected that much higher casualties would occur once their policy scores reduce to 50% of the maximum level seen in the past. Therefore, it is unwise for these countries to relax their policy as the consequences would be too much to bear.

## 7. Discussion

With a novel stochastic disease transmission model, country’s epidemic characteristics are estimated, and then used to evaluate the effectiveness of COVID-19 control strategies. The model and the estimation allow a country to obtain potential infection sizes and deaths if other country’s policy-implied epidemic characteristics were implemented in the quest for a better strategy. Our study shows that both the sizes of infection and deaths of COVID-19 are particularly responsive to early and more strict containment measures as verified not only by the results from Korea and China, but also by the potential outcomes generated under the counter-factual experiments for the USA and the UK.

There are several critical lessons one can deduce from the 25 countries’ COVID-19 experiences to prepare for the future course of the COVID-19 pandemics or other infectious disease. The first one is to take action to reduce the contact rate as early as possible, which is shown to the effective in reducing *R*_*t*_ and slowing down the infection. Taking early stringency measures is especially efficient in controlling the infection size as COVID-19 is very infectious. Second, stringency measures and economy aids are effective measures in controlling the spread of COVID-19. Our analyses shows stronger stringency measures lead to more reduction in the reproduction number and shortening the time to the turning point. The third lesson is to maintain a high level of diagnostic testing to detect and quarantine the infected cases early, which is proven successfully in Korea and by the USA and the UK diagnosis-rate-exchange experiment.

It is rather disappointing to see the second wave countries was not very responsive to the worsening epidemics in the second wave, probably due to the desire and need to re-start economy which was encouraged by the public’s fatigue to the stringency control measures and the lower death rates in many countries in the second wave. Our projections for the epidemic situations in January and February 2021 suggest that countries would have substantial epidemiological benefits if their stringency levels return to their historic maximum levels. Stronger control measures would curb the winter epidemics and avoid more deaths. There is a great urgency for those countries with low maximum stringency levels to adopt tougher containment measures than their respective maximum level in order to turn around the worsening situations.

## Data accessibility

The data are all from public data sources as stated in the Data description.
